# Supplementary material for: Identification of photoperiod-induced specific miRNAs in the adrenal glands of Sunite sheep (Ovis aries)
Source: Front Vet Sci. 2022 Jul 22;9:888207. doi: 10.3389/fvets.2022.888207 (PMC9354845; doi:10.3389/fvets.2022.888207)
Supplement: Supplementary file 1 [file Data_Sheet_1.ZIP › Supplementary file/miRDeep2 file.zip!/miRDeep2 file/Novel_Structure/SPLP42A1/Novel_431.pdf]

Provisional ID	:	NC_019467.2_15342
Score total	:	7.6
Score for star read(s)	:	3.9
Score for read counts	:	0.1
Score for mfe	:	2
Score for randfold	:	1.6
Score for cons. seed	:	
Total read count	:	12
Mature read count	:	11
Loop read count	:	0
Star read count	:	1

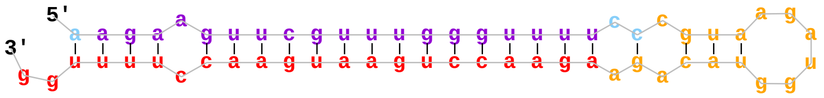

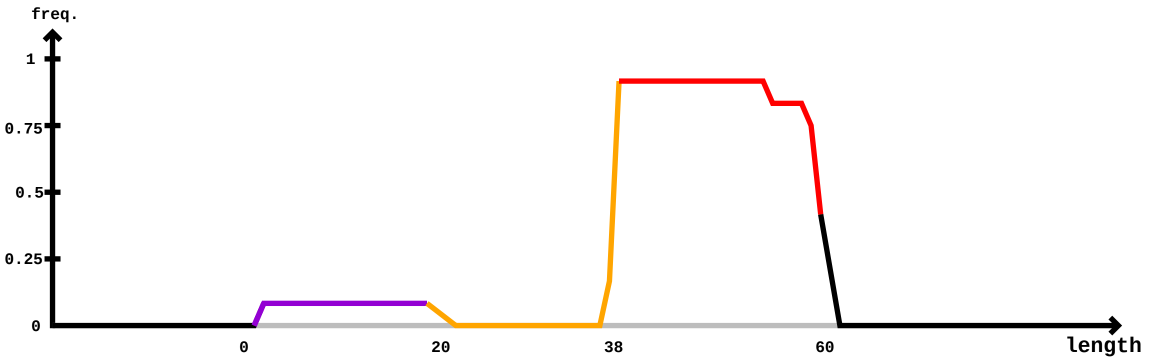


```
                    Star                                         Mature
5'- acauaauauugggugggccaagaaguucguuuggguuuucccguaagaugguacagaagaaccugaaugaaccuuuuggccagcccaguagcugcugccucuuaaucuc   -3'    obs
    acauaauauugggugggccaagaaguucguuuggguuuucccguaagaugguacagaagaaccugaaugaaccuuuuggccagcccaguagcugcugccucuuaaucuc          exp
    ......((((((((.((((((((.(((((((((((((((.(.(((......))).).))))))))))))))).)))))))).))))))))((....))...........   reads      mm         sample
    ....................agaaguucguuuggguuuu..........................................................................   1          0          seq
    ........................................................aaAaaccugaaugaacc....................................   1          1          seq
    ........................................................aaAaaccugaaugaaccuuuug...............................   1          1          seq
    .........................................................aAaaccugaaugaaccuuuu................................   1          1          seq
    .........................................................aAaaccugaaugaaccuuuug...............................   3          1          seq
    .........................................................aAaaccugaaugaaccuuuugg..............................   5          1          seq
```
